# β-caryophyllene regulates H3K36me3 to inhibit spore germination and mycelial growth of *Fusarium proliferatum*

**DOI:** 10.21203/rs.3.rs-5517661/v1

**Published:** 2025-06-24

**Authors:** Yao Zhu, Tian-Tian Li, Shi-Wei Zhou, Xue-Jie Qin, You Li, Fu-Rong Xu, Xiao-Yun Liu, Xian Dong

**Affiliations:** a School of Chinese Materia Medica, Yunnan University of Chinese Medicine, Kunming, 650500, People’s Republic of China;; b College of Life Sciences, Hubei Engineering Research Center for Protection and Utilization of Special Biological Resources in the Hanjiang River Basin/Jianghan University, Wuhan 430056, China.

**Keywords:** antifungal mechanism, *Panax notoginseng*, essential oil, plant-derived pesticides, root rot

## Abstract

**Background:**

*Panax notoginseng* is a valuable traditional Chinese medicine and is highly susceptible to root rot disease, which is primarily caused by the pathogenic fungus *Fusarium proliferatum*. The antifungal mechanisms underlying the effects of β-caryophyllene (BCP), which is commonly found in Essential oils (EOs), remain unclear. In this study, RNA sequencing (RNA-seq) and chromatin immunoprecipitation sequencing (ChIP-seq), along with in vitro antifungal experiments, were used to investigate the inhibitory effects of BCP on *F. proliferatum*.

**Results:**

BCP not only influenced the mycelial growth of *F. proliferatum* but also exerted a pronounced inhibitory effect on spore germination. After treatment with 200 mM BCP, the inhibition rate of mycelial growth was 24.5%, whereas the inhibition rate of spore germination reached 77.87%. Furthermore, after 4 h of 200 mM BCP treatment, the extracellular conductivity of *F. proliferatum* significantly increased, along with malondialdehyde content and superoxide dismutase activity, which increased to 2.6 and 1.45 times those of the control group. Transcriptome analysis revealed that, following BCP treatment, 1693 differentially expressed genes (DEGs) were upregulated and 1589 DEGs were downregulated. Additionally, BCP treatment decreased the expression of genes associated with spore germination regulation, including the transcriptional activator acu-15, serine/threonine protein phosphatases, and aldehyde dehydrogenases. Combined RNA-seq and ChIP-seq analyses revealed elevated H3K36me3 (histone H3 Lysine 36 trimethylation) modification levels in *F. proliferatum* after BCP treatment, which triggered defense mechanisms and upregulated the expression of lipid metabolism genes associated with cell membrane repair. Downregulation of the expression of genes associated with decreased H3K36me3 modification in meiosis-related and cell cycle pathways in yeast was observed, which inhibited the expression of serine/threonine protein kinase (SNF1) and the transcription factor MCM1, further suppressing spore germination and negatively impacting hyphal growth.

**Conclusions:**

This study elucidates the antifungal mechanism of BCP and provides a theoretical foundation for the development of novel plant-derived pesticides for root rot control.

## Introduction

*Panax notoginseng* (burk) F.H. Chen, a member of the Araliaceae family, is a valuable traditional Chinese medicine known for its analgesic properties and ability to enhance blood circulation [1, 2]. Additionally, *P. notoginseng* is susceptible to a variety of diseases due to its specific growth conditions and the impact of continuous cultivation practices, and root rot is the most prevalent and challenging to manage [3]. Root rot results in an average annual yield loss of 5% to 20% in *P. notoginseng*, with severe cases leading to losses as high as 70% [4]. Numerous pathogenic microorganisms contribute to root rot in *P. notoginseng*, including fungi, bacteria, and nematodes; however, fungi are the primary cause of root rot in *P. notoginseng*. The principal fungal pathogens responsible for root rot in *P. notoginseng* are *Fusarium oxysporum*, *Fusarium solani*, and *Rhizoctonia solani*. Additionally, *Fusarium proliferatum* is a significant plant pathogenic fungus capable of inducing various diseases, such as root rot, stem rot, leaf blight, and fruit spots, and it exhibits exceptionally strong pathogenicity [5].

Currently, the control of disease in *P. notoginseng* primarily relies on chemical pesticides [6]. The application of pesticides in *P. notoginseng* cultivation results in the significant accumulation of pesticide residues within the plant’s rhizome [7]. Most chemical fungicides exhibit carcinogenic and teratogenic properties, possess high toxicity, and demonstrate poor biodegradability, thereby posing serious threats to both environmental integrity and human health [8]. Consequently, there is an urgent need for an environmentally friendly approach to manage root rot in *P. notoginseng*. Plant essential oils (EOs) possess significant antimicrobial and insecticidal properties [9]. These attributes have resulted in EOs gaining considerable attention globally owing to their low residue levels, minimal potential for pollution, and overall environmental sustainability. EOs can be derived from various parts of the plant, including the flowers, leaves, stems, roots, and fruits, and consist of a mixture of volatile compounds. β-caryophyllene (BCP) is a bicyclic sesquiterpene commonly found among EOs extracted from numerous plant species. Chang et al. [10] demonstrated that BCP exhibited significant inhibitory effects against *Rhizoctonia solani* and *F. oxysporum*. Huang et al. demonstrated that the application of (*E*)-BCP on *Arabidopsis thaliana* resulted in reduced bacterial growth rates within the flower styles while simultaneously enhancing the disease resistance ability, quality, and yield of the plant [11].

Post-translational modification (PTM) of proteins plays crucial roles in the regulation of gene expression in eukaryotic organisms [12]. These modifications include phosphorylation, methylation, acetylation, and glycosylation, each of which has distinct functions and regulatory mechanisms. Methylation is prevalent in eukaryotes. In particular, H3K36me3 (histone H3 Lysine 36 trimethylation) is associated with active gene transcription. The H3K36me3 modification predominantly occurs within gene body regions and is physically associated with RNA polymerase II (RNAPII) during transcriptional elongation [13]. It is closely linked to transcriptional activity in active euchromatin processes, such as transcription initiation, DNA repair [14], and pre-mRNA splicing [15]. Adhvaryu revealed that methylation of H3K36 is essential for the normal growth and development of *Neurospora crassa* [16].

Based on the notable antimicrobial properties of EOs, the antifungal mechanisms associated with BCP, a compound frequently present in EOs, remain poorly understood. This study aimed to measure the physiological indicators of *F. proliferatum* to evaluate the antifungal effects of BCP. Additionally, we employed a combination of RNA sequencing (RNA-seq) and chromatin immunoprecipitation sequencing (ChIP-seq) analyses to elucidate the antifungal mechanisms of BCP. Based on our findings, we anticipate that further identification of the antifungal targets of BCP will provide a theoretical foundation for the development of novel plant-derived pesticides for controlling plant root rot.

## Materials and methods

### Fungal Source

The pathogenic fungus species investigated in this study was isolated and purified from roots of *P. notoginseng* exhibiting root rot. Subsequently, the purified isolate was inoculated onto potato dextrose agar (PDA) medium and incubated at 28 °C for 7 days. DNA was extracted, and the fungal internal transcribed spacer (ITS) region was amplified via PCR using primers ITS1 (5’-TCCGTAGAGGAAGTAAAAGTCG-3’) and ITS4 (5’-TCCTCCGCTTATTGATATGC-3’). Sequencing was performed by Sangon Biotech (Shanghai, China), yielding a 537-bp ITS sequence. NCBI-BLAST analysis revealed 100% identity to *Fusarium proliferatum* (MH712158.1). The sequence was deposited in GenBank under accession number OP430570.1 (*F. proliferatum*).

### Mycelial Growth Assay

The mycelial growth assay was performed following a modified protocol from Zhang M et al. [17]. BCP was dissolved in a 2% DMSO + 0.1% Tween-80 (v/v) solution (2-DMSO-T), filter-sterilized, and mixed into potato dextrose agar (PDA) medium at final concentrations of 3.125, 12.5, 50, and 200 mM. Control plates received an equivalent volume of 2-DMSO-T. Mycelial plugs (5 mm diameter) were excised from the actively growing margin of 7-day-old *F. proliferatum* colonies and centrally inoculated onto prepared PDA plates. All plates were incubated at 28°C in darkness for 5 days. Colony diameters were measured daily along two perpendicular axes and recorded from three independent biological replicates, each comprising three technical replicates. Relative growth rates were calculated as:

$$\text{Relative Growth Rate (\%)}=\frac{D_{24h}-D_{0h}}{D_{0h}}\times 100$$

where D_0h_ = initial diameter (0 h), D_24h_ = diameter after 24 h.

Fungal growth inhibition rates were calculated using the formula:

Inhibition(%)=(C−B)/C×100

where C = colony diameter of control (mm), B = colony diameter of BCP-treated group (mm). EC50 values were determined via nonlinear regression analysis (log[BCP] vs. normalized response, GraphPad Prism v9.0). Three biological replicates (each with three technical replicates) were analyzed.

### Congo Red Susceptibility Assay

To assess cell wall/membrane integrity, mycelial plugs of *F. proliferatum* were inoculated onto PDA media containing BCP (3.125–200 mM) supplemented with 200 μg/mL Congo red. Controls received equivalent volumes of 2% DMSO. Plates were incubated at 28℃for 5 days, and colony diameters were measured using the cross method. Three biological replicates (each with three technical replicates) were analyzed.

### Determination of the Spore Germination Rate

The method for determining the spore germination rate was adapted from that described by Liu et al. [18]. *F. proliferatum* was cultured for 7 days, fragmented, and subsequently transferred to Bilay’s culture medium. The BCP was dissolved in 2-DMSO-T and subsequently filtered. Thereafter, the resulting solution was added to a sterile EP tube containing 1.8 mL of 1/3 liquid PDA. The mixtures were thoroughly homogenized to obtain BCP concentrations of 3.125, 12.5, 50, and 200 mM. An equivalent volume of 2-DMSO-T served as the negative control. Subsequently, an equal amount of the spore suspension was added to attain a spore concentration of 1×10^6 spores/mL. The cultures were incubated at a constant temperature of 28 °C with shaking at 180 rpm for 14 h. For spore germination assessment, 10 μL aliquots from each spore suspension were loaded onto a hemocytometer. Observations were performed using three independent biological replicates per treatment group, with three technical replicates per biological replicate. Germinated spores were defined as those with germ tubes exceeding half the spore length. For each replicate, three random fields of view were counted, totaling ≥200 spores per treatment. Germination rates (%) were calculated as: (germinated spores / total spores) × 100%.

### Determination of the Extracellular Conductivity of Hyphae

To determine the extracellular conductivity, 1 mL of spore suspension at a concentration of 1×10^6 was added to YEPD medium and incubated at 28 °C in a shaker for 4 days. Following incubation, the mycelia were filtered, and excess water was absorbed using sterilized filter paper. BCP was dissolved in 2-DMSO-T and added to a conical flask containing 40 mL of distilled water and 2 g of fresh mycelia to create suspensions with varying concentrations (12.5, 50, and 200 mM) of BCP, with an equivalent volume of 2-DMSO-T serving as a negative control.

The extracellular conductivity of *F. proliferatum* mycelia was measured as described by Tao et al. [19]. Conductivity measurements were conducted every 2 h over a total duration of 8 h, with 2-DMSO-T used as the negative control. Extracellular conductivity was measured at each time point and expressed in microsiemens per centimeter (μs/cm). Each treatment included three independent biological replicates, with three technical replicates per biological replicate.

### Determination of Superoxide Dismutase Enzyme Activity and Malondialdehyde Content

Filtered mycelia (0.1 g) from BCP-treated samples (prepared as detailed in the section’Determination of the Extracellular Conductivity of Hyphae’) were placed in a mortar and ground in phosphate-buffered solution. The resulting mycelial extract was then centrifuged at 10,000 rpm for 15 min at 4 °C. The supernatant was collected and stored on ice until subsequent analysis.

The superoxide dismutase (SOD) activity was determined according to the method outlined by Zhou et al.[20] with minor modifications. A total of 100 μL of the mycelial extract supernatant from each treatment group was added to a series of tubes containing: 50 mM phosphate buffer, 130 mM methionine, 750 μM nitroblue tetrazolium solution, 20 μM riboflavin solution, and 100 μM EDTA-Na_2_; distilled water served as a substitute for the enzyme solution in the control group. The sample and control tubes were exposed to a fluorescent lamp emitting light at an intensity of 4000 lx at 25 °C for 18 min. Subsequently, the absorbance was measured at a wavelength of 560 nm for each treatment. Three independent biological replicates and three technical replicates per biological replicate were included. SOD activity (U/g fresh weight • h) was calculated using the formula:

$$\text{SOD activity}=\frac{\left(A_{0}-A_{n}\right)\times V\times 60}{A_{0}\times 0.5\times V_{s}\times t\times W}$$

Where:

A0: Absorbance of the control reaction mixture (without enzyme extract).

An: Absorbance of the sample reaction mixture.

V: Total volume of the extraction buffer (mL).

Vs: Volume of enzyme extract used in the assay (mL).

t: Illumination reaction time (min).

W: Fresh weight of the fungal sample (g).

Malondialdehyde (MDA) content within the *Fusarium* layer was assessed using the thiobarbituric acid (TBA) method as described by Ratnawati [21]. Specifically, 1 mL of supernatant was combined with 1 mL of a freshly prepared TBA at a concentration of 0.5%. This mixture was left to react in a boiling water bath for 10 min before being cooled and centrifuged at approximately 3000 × *g* for 15 min; TBA served as the blank control. Absorbance was measured at 450 nm (correction for sucrose interference), 532 nm (MDA-TBA adduct peak), and 600 nm (turbidity correction). Each treatment included three independent biological replicates and three technical replicates per biological replicate. MDA content (nmol/g fresh weight) was calculated using the formula:

$$\text{MDA (nmol/g FW)}=\frac{[6.452\times \left(A_{532}-A_{600}\right)-0.559\times A_{450}]\times V}{V_{s}\times W}$$

Where:

A532, A532, A600: Absorbance values at respective wavelengths.

V: Total volume of the extraction buffer (mL).

Vs: Volume of extract used in the assay (mL).

W: Fresh weight of the fungal sample (g).

### Transcriptome Sequencing

Hyphae of *F. proliferatum* were prepared using the protocol detailed in the section ‘Determination of the Extracellular Conductivity of Hyphae.’Briefly, hyphae were treated with BCP for 24 hours, and three independent biological replicates were included per treatment group. Subsequently, a low-temperature grinder was employed to pulverize the hyphae into a fine powder for further analysis. Total RNA was extracted using TRIzol reagent, and transcriptome sequencing was performed by Wuhan MetWare Biotechnology Co., Ltd. Fastp (version 0.20.0) [22] was used to filter the RNA-seq data, and low-quality sequences and adapter contamination were removed. Subsequently, DESeq2 software [23] was employed to identify significant differentially expressed genes (DEGs) between the two groups, applying screening criteria of |log2Fold Change| ≥ 1 and FDR < 0.05. Finally, functional analysis of the DEGs was performed using the Gene Ontology (GO) and KEGG (Kyoto Encyclopedia of Genes and Genomes) databases.

### Western Blotting

BCP-treated fungal powder (prepared as detailed in the section ‘Transcriptome Sequencing’) was processed using the BBproExtra^®^ Plant Histone Extraction Kit (#BB-3117) for histone extraction. Three independent biological replicates were included per treatment group to ensure statistical reliability. The primary antibodies used in the immunoblotting assays were anti-H3K36me3 (Abcam, Cat# ab9050), anti-H3 (PTM-1002) and goat anti-rabbit IgG secondary antibody (Abbkine A23220). Protein detection was performed using the ChemiDoc^™^ MP Imaging System, and ImageJ software was used to quantify the grayscale values of the bands.

### ChIP-Seq

ChIP experiments were conducted according to the method described by Zhou et al. [24]. *F. proliferatum mycelia* (2 g) treated with 200 mM BCP was ground into a powder using liquid nitrogen and cross-linked in a vacuum with 1% formaldehyde. The cross-linking reaction was terminated by the addition of glycine (2.5 M). Chromatin was extracted and fragmented by sonication to sizes ranging from 200 to 500 base pairs. ChIP was performed using anti-H3 (PTM-1002) and anti-H3K36me3 (Abcam, Cambridge, UK; Cat # ab9050) antibodies. Following the protocol provided in the Illumina TruSeq^®^ ChIP Sample Prep Set A, sequencing libraries were constructed from the ChIP DNA. Library construction and sequencing were performed at Bioacme Biotechnology Co., Ltd., using the Illumina HiSeq-PE150 platform to sequence the library products. Samtools (version 0.1.19) was used to remove potential PCR duplicates, and MACS software (version 1.4.2) was used to locate enriched regions to call Khib peaks by comparing reads from the IP sample with the input sample. Wig files produced by MACS software were used for data visualization by IGV (version 2.3.88). deepTools 2.0 software was used to generate heatmaps of different histone marks. ChIP assays were performed with three independent biological replicates per treatment group to account for biological variability and ensure robust conclusions.

### RT-qPCR and ChIP-qPCR

RT-qPCR and ChIP-qPCR were performed based on the method described by Zheng L [25] with slight modifications. Total RNA was isolated from the samples using TRIzol reagent (Transgene Biotechnology). cDNA was synthesized using ABScript Neo RT Master Mix for qPCR with gDNA Remover (RK20433). Both RT-qPCR and ChIP-qPCR were conducted in a 96-well optical plate on the CFX Connect^™^ Real-Time System (BIO-RAD) using cDNA and ChIP products as the templates and 2X Universal SYBR Green Fast qPCR Mix (RK21203) was added. The actin gene served as an internal control, and relative gene expression levels were analyzed using the 2-△△CT method [26]. The primers used are listed in Supplementary Material 1 (Tables S1 and S2).

### Statistical analysis

Statistical analyses were performed using GraphPad Prism 9 (GraphPad Software, San Diego, CA, USA). Intergroup comparisons were assessed by one-way analysis of variance (ANOVA) followed by Tukey’s post hoc test for multiple comparisons. All data are expressed as mean ± standard deviation (SD) from three independent biological replicates. A threshold of *p* < 0.05 was used to define statistical significance.

## Results

### Effects of BCP on Mycelial Growth and the Spore Germination of *F. proliferatum*

**Fig. 1:**
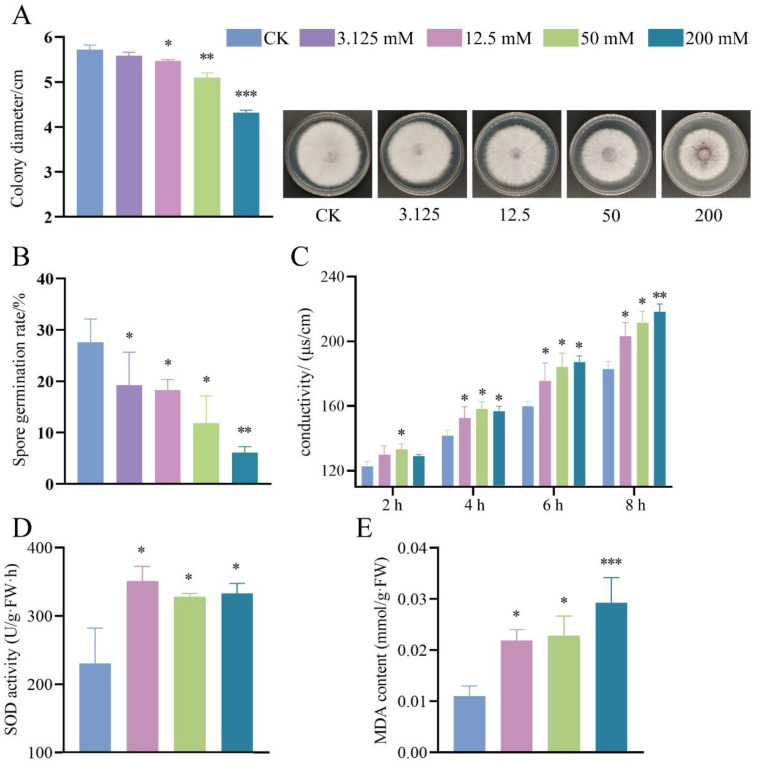
Effects of BCP on physiological and biochemical indexes of *F. proliferatum*. (A) Colony morphology after 5-day incubation with BCP (0–200 mM). (B) Spore germination rate after 14-h treatment. (C) Relative electrolyte leakage over time. (D) SOD activity and (E) MDA content after 24-h exposure. Control group (CK): 2% DMSO + 0.1% Tween-80 suspension. Data represent mean ± SD of three biological replicates (each with three technical replicates). Data are presented as mean ± SD from three biological replicates; * *p* < 0.05; ** *p* < 0.01; *** *p* < 0.001.

BCP treatment significantly inhibited the mycelial growth and spore germination of *F. proliferatum*, and the inhibitory effect increased as the BCP concentration increased. Figure 1A illustrates the growth pattern of *F. proliferatum* after 5 d. The colony diameter in the control group was 5.72 cm, while that in the 200 mM BCP treatment group was reduced to 4.3 cm, indicating a significant suppression in growth compared with that in the control group. The growth rate analysis revealed that 200 mM BCP significantly reduced both initial germination and mycelial elongation (*p* < 0.05), with treated colonies exhibiting a lower daily growth rate compared to controls (Figure S1 of Supplementary Material 1). BCP exhibited dose-dependent growth inhibition with an EC50 of 0.6 mol/L (Fig. 1A), indicating moderate antifungal efficacy under solid culture conditions. BCP-treated mycelia exhibited increased sensitivity to Congo red, with intense red staining compared to controls (Figure S2 of Supplementary Material 1), suggesting compromised cell wall/membrane integrity. After 14 hours of culture, the results presented in Fig. 1B show that at 3.125, 12.5, and 50 mM BCP, the spore germination rates were 19.27%, 18.28%, and 11.83%, respectively, which were significantly different from that in the control group (27.59%; *p* < 0.05). When the BCP concentration reached 200 mM, the spore germination rate significantly decreased to 6.11% compared with that of the control group (*p* < 0.01).

### Effects of BCP on extracellular conductivity, MDA and SOD of *F.proliferatum*

The effect of BCP on the extracellular conductivity of *F. proliferatum* is shown in Fig. 1C. Extracellular conductivity reflects membrane integrity, a critical determinant of fungal growth and survival [27]. Increased conductivity indicates compromised membrane permeability, which disrupts ion homeostasis and cellular function. MDA, a lipid peroxidation biomarker, quantifies oxidative damage to cell membranes[28]. Elevated MDA levels signify impaired antioxidant defenses and membrane destabilization. SOD activity serves as a key indicator of antioxidant capacity, counteracting reactive oxygen species (ROS) generated under stress [29]. Suppressed SOD activity suggests oxidative stress overload, further linking BCP exposure to cellular dysfunction. These parameters were selected to comprehensively evaluate BCP’s physiological impact on fungal cells. After 2 h of treatment, a significant difference in electrical conductivity was observed between the control group and the group treated with 50 mM BCP. At the 4-h mark, the groups treated with BCP concentrations of 12.5, 50, and 200 mM exhibited notable differences in electrical conductivity compared with that of the control group. Furthermore, the electrical conductivity of the solution in each treatment group gradually increased over time, leading to an increasingly pronounced difference from that of the control group. Figures 1D-E demonstrate that after treating *F. proliferatum* with 12.5, 50, and 200 mM BCP, SOD activity was significantly higher than that of the control group; specifically, SOD activity in the 12.5 mM treatment group was approximately 1.52 times that of the control group, while those in the groups treated with concentrations of 50 mM and 200 mM were approximately 1.42 and 1.45 times greater than that of the controls, respectively. Additionally, the MDA content significantly increased following treatment. The MDA levels in both groups treated with BCP at concentrations of 12.5 mM and 50 mM were approximately double those observed in the control group. In contrast, for the group treated with 200 mM BCP, MDA levels reached approximately 2.6 times that of the controls. These results indicate that BCP treatment effectively inhibited mycelial growth and spore germination in *F. proliferatum*.

### Transcriptome Analysis Following BCP Treatment

**Fig. 2:**
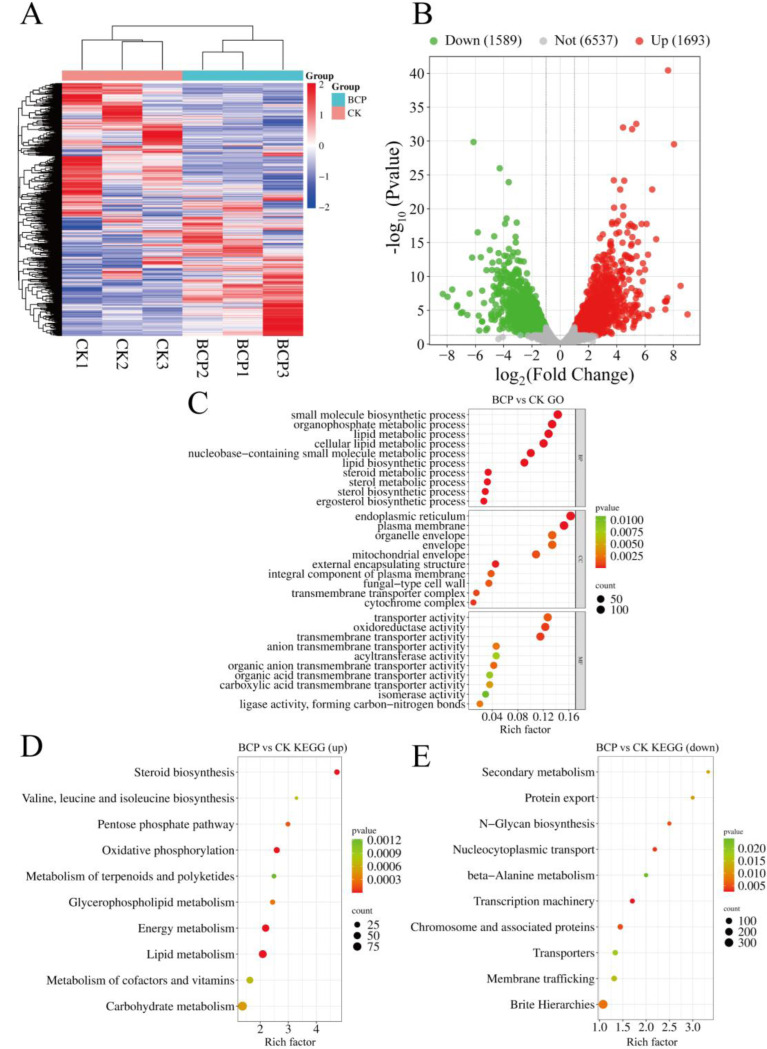
Transcriptomic analysis of *F. proliferatum* after BCP treatment. (A) Heatmap of gene expression following BCP treatment in *F. proliferatum*. (A) Heatmap of gene expression profiles. CK1-CK3: control replicates (2% DMSO + 0.1% Tween-80); BCP1-BCP3: BCP-treated replicates (200 mM). (B) Volcano plot of differentially expressed genes (DEGs) (red: upregulated; green: downregulated; gray: non-significant). (C) Top 30 enriched GO terms. BP: Biological Process; CC: Cellular Component; MF: Molecular Function. (D) Top 10 KEGG pathways for upregulated DEGs. (E) Top 10 KEGG pathways for downregulated DEGs. Enrichment ratio reflects pathway-specific downregulation. The X-axis represents the rich factor, while the Y-axis denotes pathway names. Bubble color corresponds to −log10(P-value) (gradient scale at right; darker red = higher significance), while bubble size reflects the number of downregulated DEGs per pathway.

To elucidate the molecular mechanisms by which BCP inhibited *F. proliferatum*, we conducted a transcriptome analysis of *F. proliferatum* treated with BCP. The results revealed 3,282 DEGs between the BCP-treated and control groups (CK), comprising 1,693 upregulated and 1,589 downregulated genes (Fig. 2A, B). To further investigate the functions of these DEGs in *F. proliferatum* cells, we performed GO function annotation and classification, as well as KEGG pathway enrichment analysis.

The top 30 GO terms before enrichment are shown in Fig. 2C. In the biological process category, most DEGs were enriched in the lipid, steroid, and ergosterol biosynthetic processes. In the cellular component category, the DEGs predominantly participated in pathways related to the endoplasmic reticulum, fungal-type cell wall structures, and transmembrane transporter complexes. In terms of the molecular function categories, the DEGs were primarily concentrated in oxidoreductase activity, transmembrane transporter activity, and transport activities. Sterols are intrinsically active components within biological systems that demonstrate a diverse array of biological activities, serving as essential constituents of biological membranes [30]. Our findings suggest that BCP treatment influences cell membrane synthesis by modulating sterol biosynthesis, which, in turn, alters cell membrane permeability and affects transmembrane transport in *F. proliferatum*.

KEGG pathway enrichment in the transcriptome of *F. proliferatum* treated with BCP was analyzed. The top 10 significantly upregulated and downregulated KEGG categories are shown in Fig. 2. The genes with upregulated expression were predominantly associated with pathways related to steroid biosynthesis, oxidative phosphorylation, energy metabolism, lipid metabolism, and carbohydrate metabolism (see Fig. 2D). Conversely, the genes with downregulated expression were primarily enriched in Brite Hierarchies, nucleocytoplasmic transport, transcription machinery, and chromosome and associated protein pathways (Fig. 2E). Transcriptomic analysis revealed that BCP treatment significantly downregulated genes enriched in transcriptional and metabolic pathways (e.g., Transcription machinery, beta-Alanine metabolism; Fig. 2E). These findings highlight a potential association between transcriptional/metabolic dysregulation and the observed inhibition of spore germination (19.27% at 3.125 mM vs. 27.59% in controls) and mycelial growth (4.3 cm at 200 mM vs. 5.72 cm in controls).

### Effects of BCP on Histone Methylation in *F. proliferatum*

**Fig. 3:**
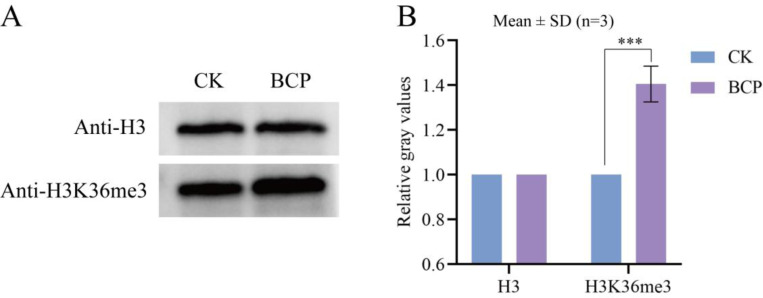
Analysis of H3K36me3 modification levels in *F. proliferatum* after BCP treatment. (A) Immunoblot of H3K36me3 (anti-H3K36me3, Abcam #ab9050) with histone H3 (PTM-1002) as a loading control. CK: Negative control (2% DMSO + 0.1% Tween-80 suspension). BCP: 200 mM BCP-treated mycelia for 24 h. Blots are representative of three independent biological replicates (See Figure S3 of supplementary material 1 for complete repetition). (B) Quantification of H3K36me3 band intensity normalized to histone H3. Data represent mean ± SD of three biological replicates (n=3). *p*< 0.001 vs. CK.

H3K36me3 is closely linked to the activation of gene expression, which involves processes such as transcription initiation and pre-mRNA splicing [15]. The findings from our study demonstrated that the level of H3K36me3 methylation in the hyphae of *F. proliferatum* was significantly elevated following BCP treatment compared with that in the control group (Fig. 3). These findings imply that BCP-induced alterations in histone methylation patterns may dysregulate the expression of growth-related genes in *F. proliferatum*, ultimately leading to fungal growth suppression.

### ChIP-seq Analysis

**Fig. 4:**
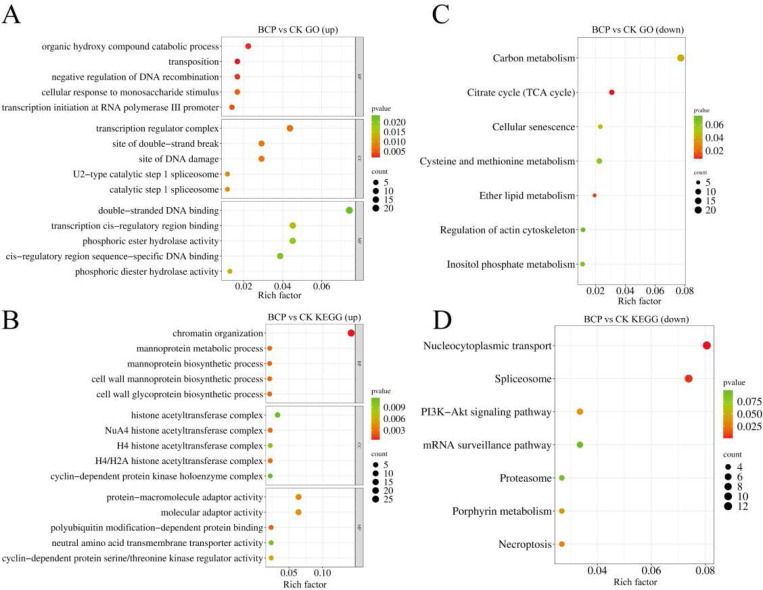
ChIP-seq analysis of *F. proliferatum* after BCP treatment. Control (CK): 2% DMSO + 0.1% Tween-80 suspension. BCP treatment: 200 mM BCP for 24 h. (A) GO enrichment for upregulated DEGs (CK vs. BCP). (B) GO enrichment for downregulated DEGs. (C) KEGG enrichment for upregulated DEGs. (D) KEGG enrichment for downregulated DEGs. Abbreviations: BP (Biological Process), CC (Cellular Component), MF (Molecular Function). Bubble plots: Color scale represents –log10 (P-value) (darker red = higher significance); bubble size corresponds to DEG count. X-axis: Enrichment ratio (up/downregulated pathways); Y-axis: pathway names.

The above results indicated that BCP treatment led to a significant increase in the abundance of histone H3K36me3. To further elucidate the regulatory mechanism of H3K36me3 in gene expression, we used an anti-H3K36me3 antibody for ChIP-seq. Compared to controls, BCP treatment induced 1,130 H3K36me3 hypermethylated peaks and 558 H3K36me3 hypomethylated peaks (Fig. 3A-B). These global methylation shifts align with our Western blot data showing reduced H3K36me3 levels (Fig. 3C), suggesting BCP may broadly disrupt histone methylation homeostasis. GO enrichment analysis revealed that the upregulated differentially modified genes (DMGs) were predominantly enriched in pathways related to the negative regulation of DNA recombination, transcription initiation by RNA polymerase III, transcriptional regulation complex binding, and binding to transcriptional cis-regulatory regions (Fig. 4A). Conversely, the downregulated DMGs were primarily associated with chromatin organization, NuA4 histone acetyltransferase complex activity, and protein-macromolecular hubbing activities (Fig. 4B).

To further validate the biological pathways affected by histone H3K36me3 modification, DMGs were subjected to KEGG enrichment analysis. The findings indicated that DEGs with upregulated expression were enriched in pathways such as the TCA cycle, cellular aging, and ether lipid metabolism (Fig. 4C), whereas downregulated DMGs were enriched in nuclear transport, mRNA surveillance pathway, spliceosome function, and PI3K-AKT signaling pathways (Fig. 4D). These results suggest that the growth inhibition of *F. proliferatum* by BCP may be associated with dysregulation of metabolic processes and nucleocytoplasmic transport, as inferred from transcriptomic and proteomic profiling.

### Combined Transcriptome and ChIP-seq Analysis

**Fig. 5:**
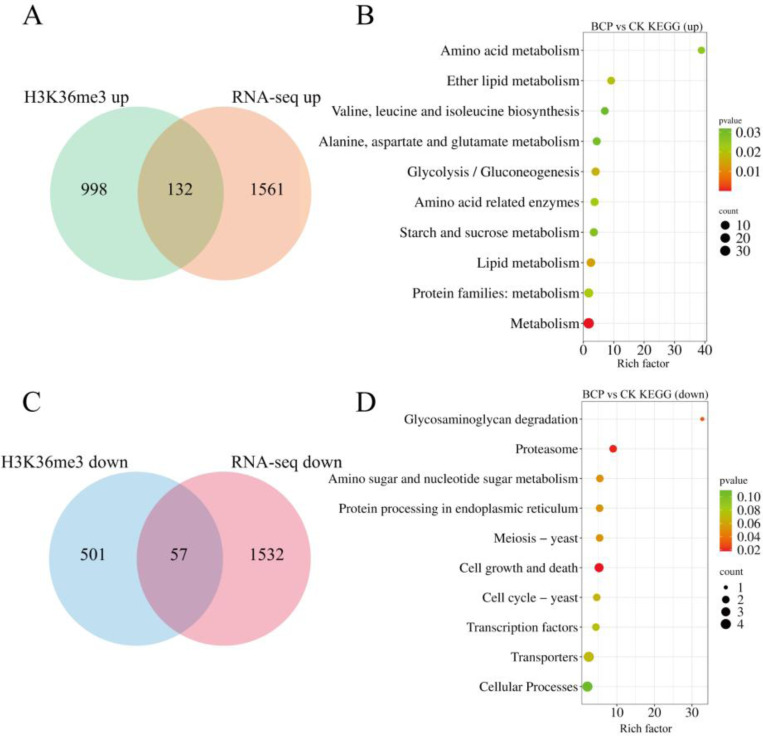
Integrated RNA-seq and ChIP-seq analysis of *F. proliferatum* after BCP treatment. (A) Venn diagram of upregulated genes overlapping between RNA-seq and H3K36me3 ChIP-seq. (B) Top 10 KEGG pathways for upregulated overlapping genes. (C) Venn diagram of downregulated genes overlapping between RNA-seq and H3K36me3 ChIP-seq. (D) Top 10 KEGG pathways for downregulated overlapping genes. Bubble plots: X-axis = Enrichment ratio (up/downregulation); Y-axis = pathway names. Color scale = –log10 (P-value); darker red = higher significance. Bubble size = number of DEGs. *p* < 0.001.

To further investigate the impact of BCP on methylation patterns in *F. proliferatum*, we performed a correlation analysis between the ChIP-seq and RNA-seq datasets. As illustrated in Fig. 5A and 5B, among the 1,130 DMGs exhibiting upregulated H3K36me3, we identified 132 commonly upregulated DEGs through RNA-seq analysis. KEGG enrichment analysis of these genes revealed significant enrichment of pathways related to metabolism, lipid metabolism, ether lipid metabolism, and energy metabolism. Similarly, as shown in Fig. 5C and 5D, within the cohort of 558 DMGs with downregulated H3K36me3 levels, we identified 57 commonly downregulated DEGs using RNA-Seq. KEGG enrichment analysis of these genes indicated significant enrichment in pathways associated with cell growth and death, meiosis (yeast), cell cycle (yeast), and transcription factors. These findings suggested that BCP enhanced the levels of H3K36me3 in *F. proliferatum*, which subsequently activated genes involved in pathways such as lipid metabolism, thereby promoting the repair of cell membrane damage. Simultaneously, BCP downregulated the expression of genes associated with the meiosis-yeast and cell cycle-yeast pathways to inhibit hyphal growth and spore germination.

Combined analysis of RNA-seq and ChIP-seq. (A) Venn diagram illustrating the number of genes with upregulated expression identified by RNA-seq and H3K36me3. (B) Top 10 KEGG pathways enriched in the genes with upregulated expression from RNA-seq and H3K36me3 analyses. (C) Venn diagram depicting the number of genes with downregulated expression identified through RNA-seq and H3K36me3 analyses. (D) Top 10 KEGG pathways enriched in the genes with downregulated expression from the RNA-seq and H3K36me3 data.

### RT-qPCR and ChIP-qPCR Analysis

**Fig. 6:**
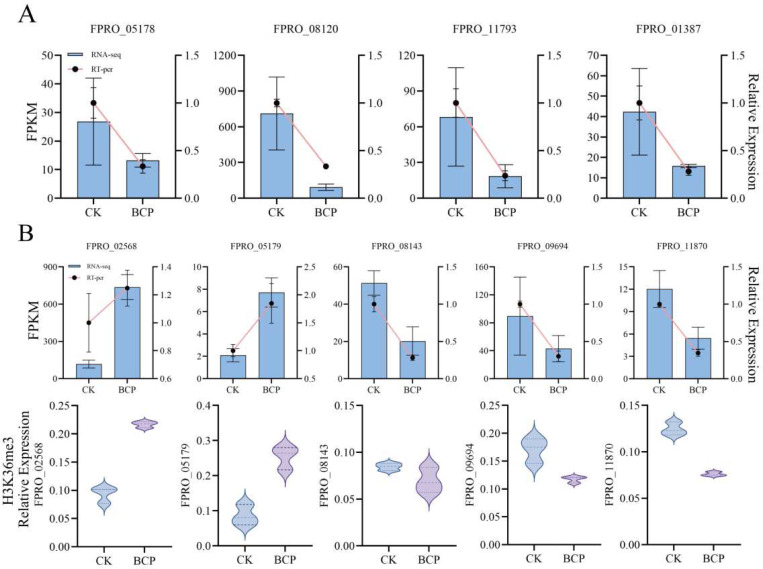
Validation of differentially expressed genes (DEGs) in *F. proliferatum* following BCP treatment. (A) RT-qPCR validation of DEGs associated with spore germination and biofilm formation. Control (CK): 2% DMSO + 0.1% Tween-80 suspension. BCP treatment: 200 mM BCP for 24 h. Left Y-axis: Transcriptomic expression levels (FPKM). Right Y-axis: RT-qPCR relative expression (2^-ΔΔCt). Error bars represent mean±SD of three biological replicates. (B) Integrated analysis of RNA-seq and H3K36me3 ChIP-seq data related to ergosterol biosynthesis and spore germination. Left Y-axis: RNA-seq expression (FPKM). Right Y-axis: RT-qPCR validation (2^-ΔΔCt). Violin plots: ChIP-qPCR validation of H3K36me3 occupancy at promoter regions of target genes. Y-axis for ChIP-qPCR: Relative enrichment (% input).

To validate the accuracy of the transcriptomic and ChIP-seq data, we compared the data for several genes using RT-qPCR and ChIP-qPCR analyses. The analyzed genes included those associated with spore germination and biofilm formation within the KEGG Brite Hierarchies pathway, as well as key components of the beta-alanine metabolism pathway identified in transcriptomic profiling: *FPRO_08120* (putative transcriptional activator acu-15), *FPRO_01387* (putative serine/threonine-protein phosphatase), *FPRO_11793* (putative NAD^⁺^-dependent aldehyde dehydrogenase ), and *FPRO_05178* (putative MRL1-related mannose 6-phosphate receptor homolog). Furthermore, through integrative analysis of ChIP-seq and RNA-seq data, we investigated genes implicated in lipid metabolism, yeast-derived meiosis/cell cycle pathways, and ergosterol biosynthesis during spore germination: *FPRO_02568* (putative squalene monooxygenase), *FPRO_05179* (putative Δ(14)-sterol reductase), *FPRO_08143* (putative putative serine/threonine protein kinase SNF1), *FPRO_09694* (putative MADS-box transcription factor MCM1), and *FPRO_11870* (putative structural maintenance of chromosomes protein). These results indicated that the trends in gene expression changes were consistent with those observed in the transcriptomic sequencing and ChIP-seq data (Fig. 6), thereby confirming the validity of our findings.

## Discussion

*F. proliferatum* is a pathogenic fungus that can induce root rot in *P. notoginseng*, bulb rot in onions, and ear rot in wheat, significantly affecting agricultural production. Recent studies have identified EOs as innovative green pesticides with remarkable antibacterial properties. BCP is a common plant-derived EO; however, the mechanism of its antifungal effect remains to be explored. In this study, we employed RNA-Seq and ChIP-Seq technologies, along with in vitro antifungal assays, to explore the inhibitory effects of BCP on *F. proliferatum* and to elucidate its mechanisms of action. The results indicated that at a concentration of 12.5 mM, BCP significantly inhibited the hyphal growth of *F. proliferatum* (Fig. 1A). Additionally, at 3.125 mM, it markedly reduced the germination rate of *F. proliferatum* spores (Fig. 1B). Furthermore, measurements of extracellular conductivity (Fig. 1C) indicated that, following BCP treatment, there was a significant increase in cell membrane permeability in *F. proliferatum* cells, which resulted in elevated SOD activity and MDA content (Fig. 1D, E), effectively impeding fungal growth. Although there is no direct observation by fluorescent probes (e.g., propidium iodide), elevated MDA levels and changes in SOD activity are recognized biomarkers of fungal oxide membrane damage [28, 29, 31]. The synergy between Congo red sensitivity (cell wall stress) and MDA/conductivity trends (membrane damage) suggests BCP simultaneously targets both structures. While Congo red assays specifically reflect ergosterol-dependent wall integrity, electrolyte leakage and lipid peroxidation are direct biomarkers of membrane rupture. This dual activity aligns with BCP’s lipophilic nature, enabling interaction with membrane lipids and indirect cell wall destabilization via ergosterol depletion. Further mechanistic studies using fluorescence microscopy will be prioritized in future work. While this study establishes BCP’s dose-responsive growth inhibition (IC50 = 0.6 mol/L), definitive classification of its fungistatic/fungicidal properties and IC90 determination require liquid culture-based assays, which are prioritized in our ongoing research.

The antimicrobial effects of natural products are frequently linked to the disruption of cell walls and membranes [32–34], inhibition of biofilm formation [35], and interference with DNA replication [36]. Analysis of the transcriptome data indicated that following treatment with BCP, steroid metabolic processes, cellular lipid metabolic processes, and ergosterol biosynthetic pathways were significantly enriched. The fungal cell membrane serves as a crucial barrier for sustaining life activities, facilitating the absorption of nutrients and the exchange of materials and energy with the external environment [37]. BCP may influence the growth of *F. proliferatum* by compromising cell membrane and wall integrity. KEGG analysis indicated that BCP treatment significantly affected the oxidative phosphorylation, energy metabolism, and carbohydrate metabolism pathways in *F. proliferatum* (Fig. 2D). Cellular metabolic activity is frequently accompanied by the generation of reactive oxygen species (ROS). Approximately 2–4% of the oxygen within the mitochondrial oxidative phosphorylation electron transport chain is converted into ROS [38, 39]. Furthermore, antifungal treatments induce ROS production in fungal cells [40]. Cell membrane integrity is essential for the maintenance of normal physiological functions in cells [41]. The accumulation of ROS within the cell can compromise the integrity of the cell membrane, resulting in lipid peroxidation, which damages the membrane. This damage further disrupts both the structure and function of the cell membrane and alters cell permeability [42]. Consequently, this inhibition affected fungal growth. In the KEGG pathway analysis of the transcriptome, we identified that the expression of *FPRO_08120* (which encodes the putative transcriptional activator acu-15), *FPRO_01387* (which encodes the putative serine/threonine protein phosphatase), and the *FPRO_11793* (which encodes the putative aldehyde dehydrogenase) within the Brite Hierarchies and beta-alanine metabolism pathway was downregulated. The decreased expression of these key proteins may directly or indirectly inhibit fungal spore germination [43–45]. Furthermore, the *FPRO_05178* gene in the Brite Hierarchies pathway contains a conserved domain associated with the autophagy-related protein ATG27. Deletion mutants of atg27 exhibit significantly reduced biofilm resistance and diminished biofilm formation [46]. The transcriptomic data suggested that BCP treatment resulted in increased permeability of the cell membrane in *F. proliferatum*, while concurrently suppressing the expression of genes associated with spore germination. These changes subsequently impacted the growth of *F. proliferatum*. While this study provides the first evidence of BCP-induced transcriptional reprogramming in *F. proliferatum*, with pathway enrichment pointing to metabolic suppression, the causal relationship between these transcriptomic changes and phenotypic inhibition remains to be fully established. Targeted genetic validation will be critical to confirm functional links. These experiments are currently prioritized in our follow-up research plan.

The compensation mechanism of fungi is a biological phenomenon wherein they activate compensatory responses to preserve the integrity of their structures and functions when exposed to external stressors. In the combined analysis of the RNA-Seq and ChIP-Seq data, DEGs that were co-upregulated by H3K36me3 modification and in the transcriptome were subjected to KEGG enrichment analysis. This analysis revealed that *FPRO_02568* (encoding squalene monooxygenase), which catalyzes the epoxidation of squalene to squalene epoxide, and *FPRO_05179* (encoding sterol reductase), both within the lipid metabolism pathway, are critical for ergosterol biosynthesis. Ergosterol is a crucial component of fungal cell membranes that regulates both membrane fluidity and permeability, while also influencing the activity of membrane-bound proteins [47]. We hypothesized that this is a possible compensatory mechanism employed by fungi in which, when external agents invade and damage cell membranes and walls, they initiate a self-protective response to upregulate the expression of ergosterol-related genes. In Bai’s research [48], eugenol treatment was found to activate bacterial enzyme antioxidant defense systems, prompting cells to regulate endogenous enzyme activity to eliminate excess ROS [49, 50]. Our findings indicated that BCP treatment resulted in increased SOD enzyme activity in *F. proliferatum*. This suggests that *F. proliferatum* adjusts its enzymatic activity to mitigate the toxic effects of these compounds.

Spore germination is essential for the survival and reproduction of fungi and plays a critical role in their normal growth and development. In the KEGG pathways enriched with DEGs exhibiting both low levels of H3K36me3 and downregulated gene expression, the expression of the *FPRO_08143*, *FPRO_09694*, and *FPRO_11870* genes, associated with the yeast meiosis and cell cycle pathways was notably repressed (Fig. 5D). *FPRO_08143* regulates the expression of serine/threonine protein kinase (SNF1) and its deletion mutant has a significantly reduced capacity for spore germination [51]. Furthermore, inhibition of SNF1 expression may lead to the failure of fungal meiosis, consequently halting growth or resulting in cell death [52]. Conversely, *FPRO_09694* modulated the expression of the transcription factor MCM1 and plays a role in positively regulating RNAPII during transcription. Knockout mutants of MCM1 exhibit defects in spore germination and are critical for maintaining cellular integrity [53, 54]. *FPRO_11870* contains a conserved structural domain characteristic of SMC proteins at its N-terminus. SMC proteins function as ATPases that preserve chromosome structure; they are essential components involved in chromosome maintenance across all living domains and are vital for cell survival [55]. Members of the SMC superfamily of ATPases contribute to chromosomal organization, dynamic maintenance processes, and DNA repair mechanisms [56]. We hypothesized that BCP treatment would decrease histone H3K36me3 modification levels. This alteration may regulate transcriptional activity, influencing RNAPII-mediated RNA synthesis, while simultaneously inhibiting fungal DNA repair processes. This leads to the suppression of spore germination, thereby impeding *F. proliferatum* growth. However, the specific mechanisms through which the aforementioned genes regulated by H3K36me3 affect the transcription process require further validation.

## Conclusion

**Fig. 7:**
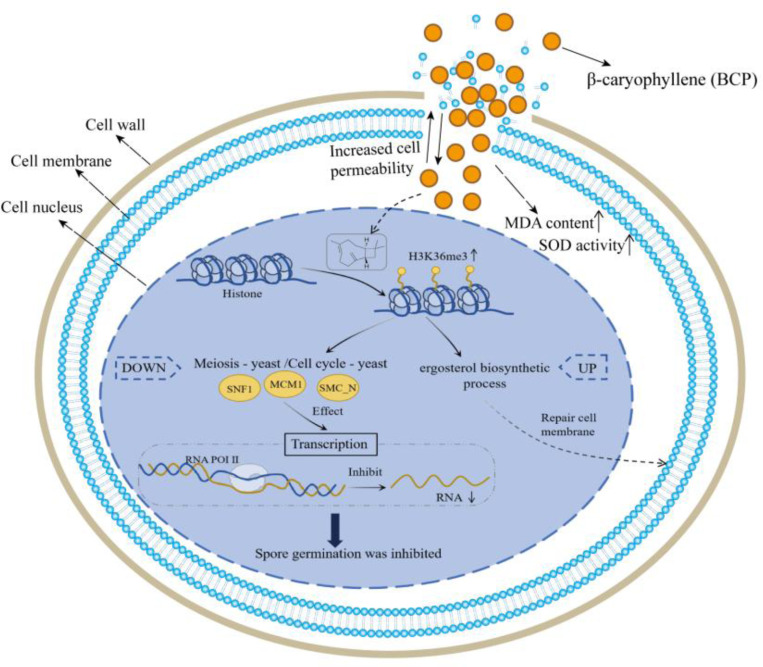
Proposed mechanism by which BCP regulates H3K36me3 to inhibit the growth and enhance fungal defense in *F. proliferatum*.

This study reveals key mechanisms underlying BCP’s antifungal effects on *F. proliferatum* (Fig. 7). BCP inhibits fungal growth by increasing membrane permeability (evidenced by electrolyte leakage, elevated MDA, and altered SOD activity), likely via lipid peroxidation. In response, *F. proliferatum* elevates H3K36me3 levels—a potential adaptive response to membrane stress—supported by upregulated ergosterol genes (*FPRO_02568/ERG1, FPRO_05179/ERG24*), though direct mechanistic ties require validation. Concurrently, BCP reduces H3K36me3 at RNAPII and DNA repair genes and dysregulates SNF1/MCM1 levels, possibly impairing transcriptional and DNA repair functions. These disruptions may collectively hinder fungal viability. Future work should validate these hypotheses through targeted gene knockouts and chromatin mapping. Overall, our findings position BCP as a promising eco-friendly antifungal agent, highlighting epigenetic and membrane targets for sustainable agriculture.

## Supplementary Material

Supplement 1

This is a list of supplementary files associated with this preprint. Click to download.

SupplementaryMaterial1.docx

SupplementaryMaterial2.xlsx

SupplementaryMaterial3.xlsx

## Data Availability

The RNA-Seq and ChIP-Seq data are accessible in the Sequence Read Archive (SRA) Database at (https://www.ncbi.nlm.nih.gov/sra). These datasets can be retrieved using the unique identifiers PRJNA1196835 for RNA-Seq and PRJNA1197138 for ChIP-Seq. A list of differentially expressed genes (DEGs) by RNA-seq and ChIP-seq, including log_2_ fold-change values, regulation directions (up/down), and functional annotations (GO/KEGG), is provided as Supplementary Material 2 (Excel format).

## References

[1] Ji C, Zhang Q, Shi R, Li J, Wang XY, Wu ZQ, Determination of the Authenticity and Origin of *Panax Notoginseng*: A Review. J AOAC Int. 2022;105(6):1708–18. 35894651 10.1093/jaoacint/qsac081

[2] Song HY, Wang PL, Liu JG, Wang CL. Panax notoginseng Preparations for Unstable Angina Pectoris: A Systematic Review and Meta-Analysis. Phytother Res. 2017;31(8):1162–72. 28634988 10.1002/ptr.5848

[3] Lorenzini G, Guidi L, Nali C, Ciompi S, Soldatini GF. Photosynthetic response of tomato plants to vascular wilt diseases. Plant Science. 1997;124(2):143–52.

[4] Liu HJ, Yang M, Zhu SS. Strategies to solve the problem of Soil sickness of Panax notoginseng (Family: Araliaceae). Allelopathy J. 2019;47(1):37–56.

[5] Yu XY, Zhang J, Guo LF, Yu AR, Wang XJ, Xiang WS, First Report of Fusarium proliferatum Causing Fruit Rot on Muskmelon (Cucumis melo) in China. Plant Dis. 2022;106(4):1. 10.1094/PDIS-09-21-2015-PDN 34645300

[6] Marschner P, Crowley D, Yang CH. Development of specific rhizosphere bacterial communities in relation to plant species, nutrition and soil type. Plant Soil. 2004;261(1–2):199–208.

[7] Zhao L, Li Y, Ren WJ, Huang Y, Wang XM, Fu ZC, Pesticide residues in soils planted with Panax notoginseng in south China, and their relationships in Panax notoginseng and soil. Ecotox Environ Safe. 2020;201:10. 10.1016/j.ecoenv.2020.110783 32534333

[8] Unnikrishnan V, Nath BS. Hazardous chemicals in foods. Indian Journal of Dairy & Biosciences. 2002.

[9] Pandey AK, Kumar P, Singh P, Tripathi NN, Bajpai VK. Essential Oils: Sources of Antimicrobials and Food Preservatives. Front Microbiol. 2017;7:14. 10.3389/fmicb.2016.02161 PMC5238431 28138324

[10] Chang HT, Cheng YH, Wu CL, Chang ST, Chang TT, Su YC. Antifungal activity of essential oil and its constituents from *Calocedrus macrolepis* var. *formosana* Florin leaf against plant pathogenic fungi. Bioresour Technol. 2008;99(14):6266–70. 18206367 10.1016/j.biortech.2007.12.005

[11] Huang M, Sanchez-Moreiras AM, Abel C, Sohrabi R, Lee S, Gershenzon J, The major volatile organic compound emitted from Arabidopsis thaliana flowers, the sesquiterpene (E)-β-caryophyllene, is a defense against a bacterial pathogen. New Phytol. 2012;193(4):997–1008. 22187939 10.1111/j.1469-8137.2011.04001.x

[12] Deribe YL, Pawson T, Dikic I. Post-translational modifications in signal integration. Nat Struct Mol Biol. 2010;17(6):666–72. 20495563 10.1038/nsmb.1842

[13] Martin C, Zhang Y. The diverse functions of histone lysine methylation. Nat Rev Mol Cell Biol. 2005;6(11):838–49. 16261189 10.1038/nrm1761

[14] Li F, Mao GG, Tong D, Huang J, Gu LY, Yang W, The Histone Mark H3K36me3 Regulates Human DNA Mismatch Repair through Its Interaction with MutSα. Cell. 2013;153(3):590–600. 23622243 10.1016/j.cell.2013.03.025 PMC3641580

[15] Sorenson MR, Jha DK, Ucles SA, Flood DM, Strahl BD, Stevens SW, Histone H3K36 methylation regulates pre-mRNA splicing in *Saccharomyces cerevisiae*. RNA Biol. 2016;13(4):412–26. 26821844 10.1080/15476286.2016.1144009 PMC4841608

[16] Adhvaryu KK, Morris SA, Strahl BD, Selker EU. Methylation of histone H3 lysine 36 is required for normal development in *Neurospora crassa*. Eukaryot Cell. 2005;4(8):1455–64. 16087750 10.1128/EC.4.8.1455-1464.2005 PMC1214527

[17] Zhang M, Li YC, Bi Y, Wang TL, Dong YP, Yang Q, 2-Phenylethyl Isothiocyanate Exerts Antifungal Activity against *Alternaria alternata* by Affecting Membrane Integrity and Mycotoxin Production. Toxins. 2020;12(2):13. 10.3390/toxins12020124 PMC7077316 32075318

[18] Liu XY, Huo YY, Yang J, Li TT, Xu FR, Wan HP, Integrated physiological, metabolomic, and proteome analysis of Alpinia officinarum Hance essential oil inhibits the growth of Fusarium oxysporum of Panax notoginseng. Front Microbiol. 2022;13:16. 10.3389/fmicb.2022.1031474 PMC9724623 36483211

[19] Tao NG, Jia L, Zhou HE. Anti-fungal activity of Citrus reticulata Blanco essential oil against *Penicillium italicum* and *Penicillium digitatum*. Food Chem. 2014;153:265–71. 24491729 10.1016/j.foodchem.2013.12.070

[20] Zhou XR, Dai L, Xu GF, Wang HS. A strain of Phoma species improves drought tolerance of Pinus tabulaeformis. Scientific Reports. 2021;11(1):7637. 33828138 10.1038/s41598-021-87105-1 PMC8027514

[21] Ratnawati D, Martono A, Fitriyanti W. Study on The Potency of Methanol Extracts From *Xanthosoma nigrum* Stellfeld As Natural Anti Oxidant by Thiobarbituric Acid Method. Aceh International Journal of Science and Technology. 2013;2(3):82–7.

[22] Chen SF, Zhou YQ, Chen YR, Gu J. fastp: an ultra-fast all-in-one FASTQ preprocessor. Bioinformatics. 2018;34(17):884–90. 30423086 10.1093/bioinformatics/bty560 PMC6129281

[23] Love MI, Huber W, Anders S. Moderated estimation of fold change and dispersion for RNA-seq data with DESeq2. Genome Biol. 2014;15(12):38. 10.1186/s13059-014-0550-8 PMC4302049 25516281

[24] Zhou C, Zhou HL, Ma XP, Yang HL, Wang P, Wang GD, Genome-Wide Identification and Characterization of Main Histone Modifications in Sorghum Decipher Regulatory Mechanisms Involved by mRNA and Long Noncoding RNA Genes. J Agric Food Chem. 2021;69(7):2337–47. 33555853 10.1021/acs.jafc.0c07035

[25] Zheng LL, Li C, Ma XP, Zhou HL, Liu Y, Wang P, Functional interplay of histone lysine 2-hydroxyisobutyrylation and acetylation in Arabidopsis under dark-induced starvation. Nucleic Acids Res. 2021;49(13):7347–60. 34165567 10.1093/nar/gkab536 PMC8287917

[26] Livak KJ, Schmittgen TD. Analysis of relative gene expression data using real-time quantitative PCR and the 2-ΔΔCT method. Methods. 2001;25(4):402–8. 11846609 10.1006/meth.2001.1262

[27] Nie HY, Liao HX, Wen JR, Ling CQ, Zhang LY, Xu FR, Foeniculum vulgare essential oil nanoemulsion inhibits Fusarium oxysporum causing Panax notoginseng root-rot disease. J Ginseng Res. 2024;48(2):236–44. 38465211 10.1016/j.jgr.2023.12.002 PMC10920008

[28] Zhao YT, Wang XE, Zhang L, Wang KY, Wu YC, Yao J, Anti-Fungal Activity of Moutan cortex Extracts against Rice Sheath Blight (Rhizoctonia solani) and Its Action on the Pathogen’s Cell Membrane. ACS Omega. 2022;7(50):47048–55. 36570206 10.1021/acsomega.2c06150 PMC9773796

[29] Li N, Wu YX, Zhang YD, Wang SR, Zhang GC, Yang J. Phytic acid is a new substitutable plant-derived antifungal agent for the seedling blight of Pinus sylvestris var. mongolica caused by Fusarium oxysporum. Pest Biochem Physiol. 2023;191:11. 10.1016/j.pestbp.2023.105341 36963923

[30] Depta L, Whitmarsh-Everiss T, Laraia L. Structure, function and small molecule modulation of intracellular sterol transport proteins. Bioorg Med Chem. 2022;68:12. 10.1016/j.bmc.2022.116856 35716590

[31] Ma DC, Wang GX, Zhu JM, Mu W, Dou DL, Liu F. Green Leaf Volatile *Trans*-2-Hexenal Inhibits the Growth of *Fusarium graminearum* by Inducing Membrane Damage, ROS Accumulation, and Cell Dysfunction. J Agric Food Chem. 2022;70(18):5646–57. 35481379 10.1021/acs.jafc.2c00942

[32] Cui HY, Bai M, Sun YH, Abdel-Sarnie MAS, Lin L. Antibacterial activity and mechanism of Chuzhou chrysanthemum essential oil. J Funct Food. 2018;48:159–66.

[33] Hu W, Li CZ, Dai JM, Cui HY, Lin L. Antibacterial activity and mechanism of Litsea cubeba essential oil against methicillin-resistant Staphylococcus aureus (MRSA). Industrial Crops and Products. 2019;130:34–41.

[34] Wongsawan K, Chaisri W, Tangtrongsup S, Mektrirat R. Bactericidal Effect of Clove Oil against Multidrug-Resistant Streptococcus suis Isolated from Human Patients and Slaughtered Pigs. Pathogens. 2020;9(1):12. 10.3390/pathogens9010014 PMC7169397 31877814

[35] Latifah-Munirah B, Himratul-Aznita WH, Zain NM. Eugenol, an essential oil of clove, causes disruption to the cell wall of Candida albicans (ATCC 14053). Front Life Sci. 2015;8(3):231–40.

[36] Rajkowska K, Otlewska A, Kunicka-Styczynska A, Krajewska A. *Candida albicans* Impairments Induced by Peppermint and Clove Oils at Sub-Inhibitory Concentrations. Int J Mol Sci. 2017;18(6):11. 10.3390/ijms18061307 PMC5486128 28629195

[37] Sant DG, Tupe SG, Ramana CV, Deshpande MV. Fungal cell membrane-promising drug target for antifungal therapy. J Appl Microbiol. 2016;121(6):1498–510. 27667746 10.1111/jam.13301

[38] Migliaccio E, Giorgio M, Pelicci PG. Apoptosis and aging: Role of p66^Shc^ Redox protein. Antioxid Redox Signal. 2006;8(3–4):600–8. 16677103 10.1089/ars.2006.8.600

[39] Giorgio M, Migliaccio E, Orsini F, Paolucci D, Moroni M, Contursi C, Electron transfer between cytochrome c and p66^Shc^ generates reactive oxygen species that trigger mitochondrial apoptosis. Cell. 2005;122(2):221–33. 16051147 10.1016/j.cell.2005.05.011

[40] Lee Y, Puumala E, Robbins N, Cowen LE. Antifungal Drug Resistance: Molecular Mechanisms in *Candida albicans* and Beyond. Chem Rev. 2021;121(6):3390–411. 32441527 10.1021/acs.chemrev.0c00199 PMC8519031

[41] Lin YF, Lin YX, Lin HT, Shi J, Chen YH, Wang H. Inhibitory effects of propyl gallate on membrane lipids metabolism and its relation to increasing storability of harvested longan fruit. Food Chem. 2017;217:133–8. 27664618 10.1016/j.foodchem.2016.08.065

[42] Blanch M, Alvarez I, Sanchez-Ballesta MT, Escribano MI, Merodio C. Involvement of fatty acids in the response to high CO2 and low temperature in harvested strawberries. Postharvest Biol Technol. 2019;147:196–205.

[43] Zhang Y, Zhuang XY, Meng JX, Zan FF, Liu ZR, Qin CC, A Putative Zn(II)2Cys6-Type Transcription Factor FpUme18 Is Required for Development, Conidiation, Cell Wall Integrity, Endocytosis and Full Virulence in Fusarium pseudograminearum. Int J Mol Sci. 2023;24(13):13. 10.3390/ijms241310987 PMC10341630 37446163

[44] Huang Z, Cao H, Wang H, Huang P, Wang J, Cai YY, The triglyceride catabolism regulated by a serine/threonine protein phosphatase, Smek1, is required for development and plant infection in *Magnaporthe oryzae*. Molecular Plant Pathology. 2023;24(10):1256–72. 37357820 10.1111/mpp.13368 PMC10502837

[45] Tang L, Zhai HC, Zhang SB, Lv YY, Li YQ, Wei S, Functional Characterization of Aldehyde Dehydrogenase in Fusarium graminearum. Microorganisms. 2023;11(12):20. 10.3390/microorganisms11122875 PMC10745421 38138019

[46] Liu SQ, Jiang LL, Miao HC, Lv Y, Zhang QQ, Ma M, Autophagy regulation of ATG13 and ATG27 on biofilm formation and antifungal resistance in Candida albicans. Biofouling. 2022;38(9):926–39. 36476055 10.1080/08927014.2022.2153332

[47] Wollam J, Antebi A. Sterol Regulation of Metabolism, Homeostasis, and Development. In: Kornberg RD, Raetz CRH, Rothman JE, Thorner JW, editors. Annual Review of Biochemistry, Vol 80. Palo Alto: Annual Reviews; 2011. p. 885–916. 10.1146/annurev-biochem-081308-165917 PMC3918218 21495846

[48] Bai JW, Li JQ, Chen ZY, Bai XD, Yang ZY, Wang ZT, Antibacterial activity and mechanism of clove essential oil against foodborne pathogens. LWT-Food Sci Technol. 2023;173:9.

[49] Cheng LZ, Wang YF, He XX, Wei XL. Preparation, structural characterization and bioactivities of Se-containing polysaccharide: A review. Int J Biol Macromol. 2018;120:82–92. 30114426 10.1016/j.ijbiomac.2018.07.106

[50] Lin YL, Tang X, Xu LZ, Wang SY. Antibacterial properties and possible action mechanism of chelating peptides-zinc nanocomposite against Escherichia coli. Food Control. 2019;106:8.

[51] Feng J, Zhang H, Strelkov SE, Hwang SF. The *LmSNF1* Gene Is Required for Pathogenicity in the Canola Blackleg Pathogen *Leptosphaeria maculans*. PLoS One. 2014;9(3):11. 10.1371/journal.pone.0092503 PMC3956939 24638039

[52] Purnapatre K, Piccirillo S, Schneider B, Honigberg S. The *CLN3/SWI6/CLN2* pathway and *SNF1* act sequentially to regulate meiotic initiation in *Saccharomyces cerevisiae*. Genes to Cells. 2010;7(7):675–91. 10.1046/j.1365-2443.2002.00551.x 12081645

[53] Yang C, Liu HQ, Li GT, Liu MG, Yun YZ, Wang CF, The MADS-box transcription factor FgMcm1 regulates cell identity and fungal development in *Fusarium graminearum*. Environ Microbiol. 2015;17(8):2762–76. 25627073 10.1111/1462-2920.12747

[54] Zhao X, Yang XJ, Lu ZY, Wang HF, He ZJ, Zhou GY, MADS-box transcription factor Mcm1 controls cell cycle, fungal development, cell integrity and virulence in the filamentous insect pathogenic fungus *Beauveria bassiana*. Environ Microbiol. 2019;21(9):3392–416. 30972885 10.1111/1462-2920.14629

[55] Losada A, Hirano T. Dynamic molecular linkers of the genome: the first decade of SMC proteins. Genes Dev. 2005;19(11):1269–87. 15937217 10.1101/gad.1320505

[56] Yoshinaga M, Nakayama T, Inagaki Y. A novel structural maintenance of chromosomes (SMC)-related protein family specific to Archaea. Front Microbiol. 2022;13:9. 10.3389/fmicb.2022.913088 PMC9389158 35992648

