# Supplementary material for: β-caryophyllene regulates H3K36me3 to inhibit spore germination and mycelial growth of Fusarium proliferatum
Source: Res Sq. 2025 Jun 24:rs.3.rs-5517661. Preprint. [Version 1] doi: 10.21203/rs.3.rs-5517661/v1 (PMC12270221; doi:10.21203/rs.3.rs-5517661/v1)
Supplement: Supplement 1 [file NIHPPRS5517661V1-supplement-1.pdf]

Supplementary material

Supplementary Material 1:

Figure S1. Growth rate comparison of *F. proliferatum* under 200 mM BCP treatment vs. control (n= 5 d cultivation). Control group (CK): 2% DMSO + 0.1% Tween-80 suspension. Data represent mean ± SD of three biological replicates (each with three technical replicates). Data are presented as mean ± SD from three biological replicates; * $p < 0.05$; ** $p < 0.01$.

Figure S2. The colony morphology of *F. proliferatum* treated with different concentrations of BCP for 5 days in Congo red medium. Control group (CK): 2% DMSO + 0.1% Tween-80 suspension. Data represent mean ± SD of three biological replicates (each with three technical replicates). Data are presented as mean ± SD from three biological replicates; * $p < 0.05$; ** $p < 0.01$; *** $p < 0.001$.

Figure S3. Three independent biological replicates of Western blotting. (A, B, C) Immunoblot analysis of anti-H3 levels in *F. proliferatum*. A, B and C are three biological replicates. (D, E, F) Immunoblot analysis of anti-H3K36me3 levels in *F. proliferatum*. D, E and F are three biological replicates. Figure 3A in the revised manuscript is cropped from A and D.

Table S1. Primers for RT-qPCR.

Table S2. Primers for ChIP-qPCR.

Supplementary Material 2: The complete list of differentially expressed genes (DEGs) of RNA-seq, including log_2 fold-change values, regulation directions (up/down), and

functional annotations (GO/KEGG).

Supplementary Material 3: The complete list of differentially expressed genes (DEGs) of ChIP-seq, including log_2 fold-change values, regulation directions (up/down), and functional annotations (GO/KEGG).
